# Comparison of cyclic RGD peptides for α_v_β_3_ integrin detection in a rat model of myocardial infarction

**DOI:** 10.1186/2191-219X-3-38

**Published:** 2013-05-11

**Authors:** Iina Laitinen, Johannes Notni, Karolin Pohle, Martina Rudelius, Eliane Farrell, Stephan G Nekolla, Gjermund Henriksen, Stefanie Neubauer, Horst Kessler, Hans-Jürgen Wester, Markus Schwaiger

**Affiliations:** 1 Department of Nuclear Medicine, Klinikum rechts der Isar, Technische Universität München, Ismaninger Strasse 22, Munich 81675, Germany; 2 Pharmaceutical Radiochemistry, Technische Universität München, Walther-Meissner-Str. 3, Garching 85748, Germany; 3 Institute of Pathology, Technische Universität München, Ismaningerstrasse 22, Munich 81675, Germany; 4 Department of Chemistry, Institute for Advanced Study (IAS) and Center of Integrated Protein Science (CIPSM), Technische Universität München, Lichtenbergstrasse 4, Garching 85748, Germany; 5 Chemistry Department, Faculty of Science, King Abdulaziz University, P.O. Box 80203, Jeddah 21589, Saudi Arabia

**Keywords:** ^18^F-galacto-RGD, ^68^Ga-NODAGA-RGD, ^68^Ga-TRAP(RGD)_3_, PET, Myocardial infarction

## Abstract

**Background:**

Expression of α_v_β_3_ integrin is increased after myocardial infarction as part of the repair process. Increased expression of α_v_β_3_ has been shown by molecular imaging with ^18^F-galacto-RGD in a rat model. The ^68^Ga-labelled RGD compounds ^68^Ga-NODAGA-RGD and ^68^Ga-TRAP(RGD)_3_ have high specificity and affinity, and may therefore serve as alternatives of ^18^F-galacto-RGD for integrin imaging.

**Methods:**

Left coronary artery ligation was performed in rats. After 1 week, rats were imaged with [^13^N]NH_3_, followed by ^18^F-galacto-RGD, ^68^Ga-NODAGA-RGD or ^68^Ga-TRAP(RGD)_3_ using a dedicated animal PET/CT device. Rats were killed, and the activity in tissues was measured by gamma counting. The heart was sectioned for autoradiography and histology. Immunohistochemistry was performed on consecutive sections using CD31 for the endothelial cells and CD61 for β_3_ expression (as part of the α_v_β_3_ receptor).

**Results:**

*In vivo* imaging showed focal RGD uptake in the hypoperfused area of infarcted myocardium as defined with [^13^N]NH_3_ scan. In autoradiography images, augmented uptake of all RGD tracers was observed within the infarct area as verified by the HE staining. The tracer uptake ratios (infarct vs. remote) were 4.7 ± 0.8 for ^18^F-galacto-RGD, 5.2 ± 0.8 for ^68^Ga-NODAGA-RGD, and 4.1 ± 0.7 for ^68^Ga-TRAP(RGD)_3_. The ^68^Ga-NODAGA-RGD ratio was higher compared to ^68^Ga-TRAP(RGD)_3_ (*p* = 0.04), but neither of the ^68^Ga tracers differed from ^18^F-galacto-RGD (*p* > 0.05). The area of augmented ^68^Ga-RGD uptake was associated with β_3_ integrin expression (CD61).

**Conclusion:**

^68^Ga-NODAGA-RGD and ^68^Ga-TRAP(RGD)_3_ uptake was equally increased in the infarct area at 1 week post infarction as ^18^F-galacto-RGD. These results show the potential of ^68^Ga-labelled RGD peptides to monitor integrin expression as a part of myocardial repair and angiogenesis after ischaemic injury *in vivo*.

## Background

Healing of myocardial infarct (MI) is a dynamic process, with stages of initial inflammation, angiogenesis, fibroblast proliferation and collagen deposition followed by scar formation in the maturation and remodelling phase [1]. Inadequately healed MI results in infarct area expansion and dilatation of the heart by left ventricle (LV) remodelling, ultimately developing into chronic heart failure [2,3]. Angiogenesis is a central part of infarct healing and is characterised by activation of angiogenic factors and expression of signalling molecules such as integrins. One particularly interesting member of this family is the integrin α_v_β_3_. It is a cell-membrane glycoprotein receptor that is quiescent under normal conditions, but its expression and activation is up-regulated on the endothelial cells when angiogenesis takes place, such as in the infarct area after ischaemic myocardial injury [4]. Radiolabelled antagonists containing the α_v_β_3_-specific cyclic RGD peptide can be used for molecular imaging of α_v_β_3_ integrin expression after MI [5-7]. Uptake is the highest at 1 week after infarct corresponding to the peak time of neovessel formation [5]. Our recent study shows that the uptake of ^18^F-galacto-cyclo(RGDfK) (^18^F-galacto-RGD) in the infarct area at 1 week after MI predicts improved healing in the form of less LV remodelling seen 12 weeks after the injury [8]. Taken together, imaging of α_v_β_3_ integrin expression is an attractive tool to assay the post-MI angiogenesis and may be potentially used to predict outcome of infarct healing.

The production of ^18^F-galacto-RGD requires an on-site cyclotron and a multi-step synthesis, which is challenging to transfer to good manufacturing practice (GMP) conditions. Therefore, alternative RGD compounds have been sought after, such as a one-step-labelled positron-emission tomography (PET) tracer,^18^F-AIF-NOTA-PRGD_2_, which was also found to reflect ischaemia-induced angiogenesis in a rat model [7].^68^Ga-NODAGA-cyclo(RGDyK) and ^68^Ga-TRAP(cyclo(RGDfK))_3_ have been evaluated for α_v_β_3_ imaging in tumour models, showing high target-to-background ratios [9-12]. ^68^Ga tracers have the advantage of easy and fast production with a generator-produced radionuclide. ^68^Ga-NODAGA-RGD and particularly ^68^Ga-TRAP(RGD)_3_ can be produced with high specific activity (approximately 200 to 600 GBq/μmol for ^68^Ga-NODAGA-RGD and over 5 TBq/μmol for ^68^Ga-TRAP(RGD)_3_[9,11]), which is essential when using ^68^Ga-labelled peptides, especially in case of small animal studies.

In this study we sought to evaluate ^68^Ga-NODAGA-RGD and ^68^Ga-TRAP(RGD)_3_ for targeting α_v_β_3_ integrin in comparison to ^18^F-galacto-RGD using a well-defined rat model of myocardial infarct [6,8]. We investigated the uptake of all three tracers using *in vivo* and *ex vivo* techniques and validated the findings with immunohistochemistry.

## Methods

### Animal model

Permanent ligation of the left anterior descending coronary artery was performed, as previously described [8] in 34 healthy male Lewis rats (Charles Rivers Laboratories, Sulzfeld, Germany) aged 8 to 10 weeks to induce MI. Briefly, the rats were anaesthetised with intramuscular administration of midazolam 5 mg/kg (Dormicum®, Roche; Grenzach-Wyhlen, Germany), medetomidin 0.5 mg/kg (Dormitor®, Pfizer, Karlsruhe, Germany) and fentanyl 0.05 mg/kg (Ratiopharm, Ulm, Germany) and connected to a rodent ventilator. The heart was exposed through a left lateral thoracotomy of the fourth intercostal space, and the left anterior descending (LAD) artery was ligated near to its origin (2 to 3 mm from the tip of the left atrium). Two rats underwent sham operation, which consisted of the same procedures except that the LAD artery suture was not tightened. The rats were treated with 0.05 mg/kg buprenorphin (Temgesic®, Essex, Germany) and 4 mg/kg carprofen (Rimadyl, Pfizer, Germany) for 72 h post surgery. The study protocols were approved by the regional governmental commission for animal protection (Regierung von Oberbayern, Munich, Germany).

### Tracer preparation

The radiosynthesis and quality control of ^18^F-galacto-RGD (^18^F-galacto-cyclo(RGDfK)), ^68^Ga-NODAGA-RGD (^68^Ga-NODAGA-cyclo(RGDyK)) and ^68^Ga-TRAP(RGD)_3_ (^68^Ga-TRAP(cyclo(RGDfK))_3_) were performed as described previously [9-12].

### Uptake of RGD in tissues

Rats were anesthetised using 1.5% isoflurane and were injected via tail vein catheter with ^18^F-galacto-RGD (37 MBq, *n* = 9), ^68^Ga-NODAGA-RGD (69 MBq, *n* = 10) or of ^68^Ga-TRAP(RGD)_3_ (54 MBq, *n* = 12). Rats were kept under anaesthesia in a warmed bed until euthanized after 90 min using intravenous injection of 150 mg/kg of pentobarbiturate (Narcoren®, Merial, Rohrdorf, Germany). The heart was excised and cut transaxially at the location of the infarct. The apical part, containing both infarct and non-affected remote myocardium, was processed for sectioning for autoradiography as described below.

Tissue samples (remote myocardium, apical part of myocardium, femoral muscle, operation wound and the connective tissue contralateral to the wound, spleen, liver, kidney, urine and blood) were dissected, and the weights of the tissue, urine, blood and serum samples were measured and their activity was detected using a gamma counter (1480 Wizard, PerkinElmer Wallac, Turku, Finland).

In order to determine the blood clearance, blood samples were drawn from a subset of animals (^68^Ga-NODAGA-RGD, *n* = 3; ^68^Ga-TRAP(RGD)_3_, *n* = 3) at 1, 5 and every 10 min after injection using a second tail vein catheter.

The specificity of the RGD binding was studied on three rats with pre-injection of a blocking dose of cilengitide (18 mg/kg = 30.6 μmol/kg) 10 min prior to the tracer injection [12-15].

### Autoradiography

The excised hearts were frozen and embedded in Tissue Tek® mounting media (Sakura Finetek Europe B.V., Alphen aan den Rijn, the Netherlands). Serial LV short-axis cryosections of 20-μm thickness were obtained. Similar sections of muscle and spleen samples of the same rat were processed and used as negative and positive controls. After quick air drying, the sections were exposed to an imaging plate (Kodak Storage Phosphor screen GP, Eastman Kodak Company, Rochester, NY, USA). After an overnight exposure, the imaging plates were scanned with an image plate scanner (CR 35 BIO, Dürr Medical, Raytest Isotopenmeßgeräte GmbH, Germany; internal resolution of 25 μm), and the images were analysed for background-corrected count densities (PSL/mm^2^) with an image analysis programme (AIDA Image Analyser, Raytest Isotopenmeßgeräte, Germany). Later, the same sections were stained with hematoxylin-eosin (HE) for delineation of the affected area in the myocardium.

After careful co-registration of the autoradiographs and HE histological images, regional tracer uptake was analysed in the following two regions: (1) the infarcted myocardium containing the border zone as assessed after HE staining and (2) the remote, non-affected myocardium. The analysis was based on 12 to 15 sections from each heart. The results were expressed as a ratio of the mean value of activity in the infarct area divided by the mean value of activity in the remote area.

### PET imaging

A subset of animals (*n* = 7) was imaged using a small-animal PET/CT scanner (Inveon, Siemens Medical Solutions, Knoxville, TN, USA). The rats were anaesthetised using 1.5% isoflurane. Breathing was monitored and temperature was maintained using a heating pad throughout the imaging procedures. The rats were injected with 37 MBq of [^13^N]NH_3_ via tail a vein catheter, and perfusion images were acquired for 15 min starting at the time of injection. A CT scan was performed for anatomical localisation. The animals were left anaesthetised in the scanner, and 60 min later, the animals were injected with either ^68^Ga-NODAGA-RGD (72 MBq, *n* = 4) or ^68^Ga-TRAP(RGD)_3_ (63 MBq, *n* = 3) via the tail vein. Dynamic PET data were acquired from the start of the injection for 90 min in a list mode. The rats were euthanized immediately after, and the tissues were processed as described above.

PET data were reconstructed using a 3D-filtered back-projection algorithm. The resulting matrix was 128 × 128 pixels with 159 transverse slices (voxel size of 0.78 × 0.78 × 0.80 mm^3^). Data were normalised and corrected for randoms, dead time and decay. No corrections were made for attenuation or scatter.

The CT acquisition consisted of 120 projections acquired with exposure time of 200 ms, X-ray voltage of 80 kVp and anode current of 500 μA for 220° rotation. CT images were reconstructed using a modified Feldkamp algorithm. The resulting matrix was 256 × 256 pixels with 631 transverse slices (reconstructed voxel size 0.21 × 0.21 × 0.21 mm^3^).

The ^68^Ga- and ^13^N-PET images were fused, and the uptake was visualised using the Inveon Research Workplace (Siemens, Knoxville, TN, USA). PET and CT images were co-registered for anatomical reference. Regions of interest (ROI) were manually drawn around the focal ^68^Ga-RGD uptake area in the myocardium on a transverse image, corresponding to ^13^N signal loss. This was repeated on four consecutive transverse images to cover the infarct area. The mean radioactivity concentration within the ROI was expressed as the percentage of injected dose per cubic centimetre (% ID/cc).

### Immunohistochemistry

Five-micron LV short-axis sections were processed adjacent to the plane of sections for autoradiography. To visualise the endothelial cells, macrophages and β_3_ integrin expression in the myocardium, sections were stained with the following antibodies: CD31 (BD Pharmingen, Franklin Lakes, NJ, USA), F4/80 (Abcam, Cambridge, UK) and CD61 (BD Pharmingen, Franklin Lakes, NJ, USA). The sections were analysed for the extent of positive staining in remote, borderzone and infarct by light microscopy at ×400 magnification. After averaging the positive counts of ten fields, the vascular density and macrophage and β_3_ integrin expression were calculated per area (high-power field). For correlation with RGD uptake in the autoradiograms, the borderzone and infarct area results were pooled.

### Statistical analysis

All data are expressed as mean ± standard deviation. The *p* values of <0.05 were considered as statistically significant. For comparison between two groups, Student's *t* test for unpaired data was used. ANOVA with Tukey-Kramer adjustment was used for comparisons of three different groups. For the correlation between two continuous variables, linear regression with Pearson's or Spearman's (for non-parametric) rank test was used.

## Results

### Animal model

Ligation of the LAD resulted in a myocardial infarction, which covered 15% to 25% of myocardial axial plain, as measured from autoradiography sections. Out of 34 operated rats, two died within 24 h after surgery and one during the imaging day, and one had 100% infarct in cross section and was therefore excluded. Average body weights were 310 ± 54 g and did not differ between the groups.

**Figure 1:**
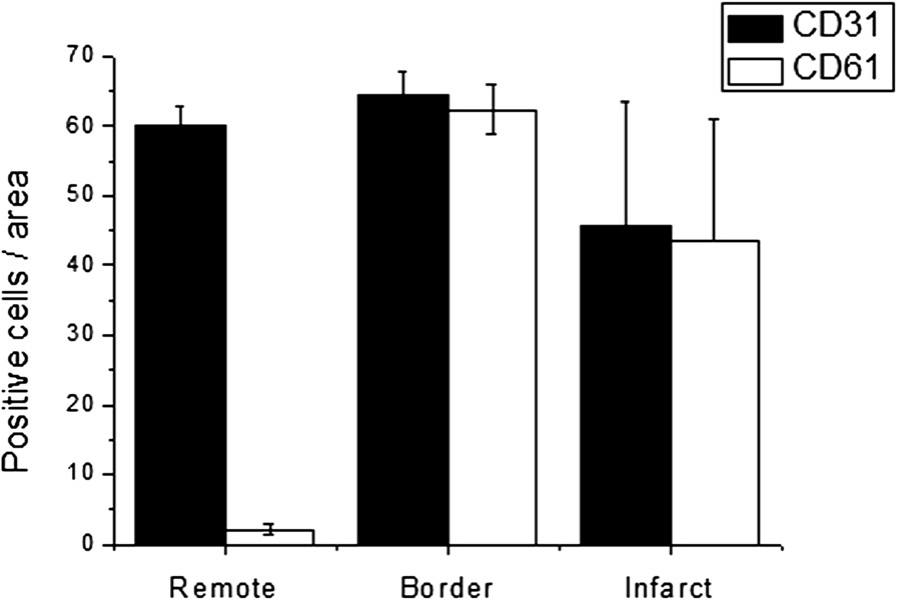
**Vascular density (CD31) and β**_**3 **_**integrin expression (CD61) in remote, borderzone and infarct areas.** Average ± SD of positive counts of 10 high power field (×400 magnification).

**Figure 2:**
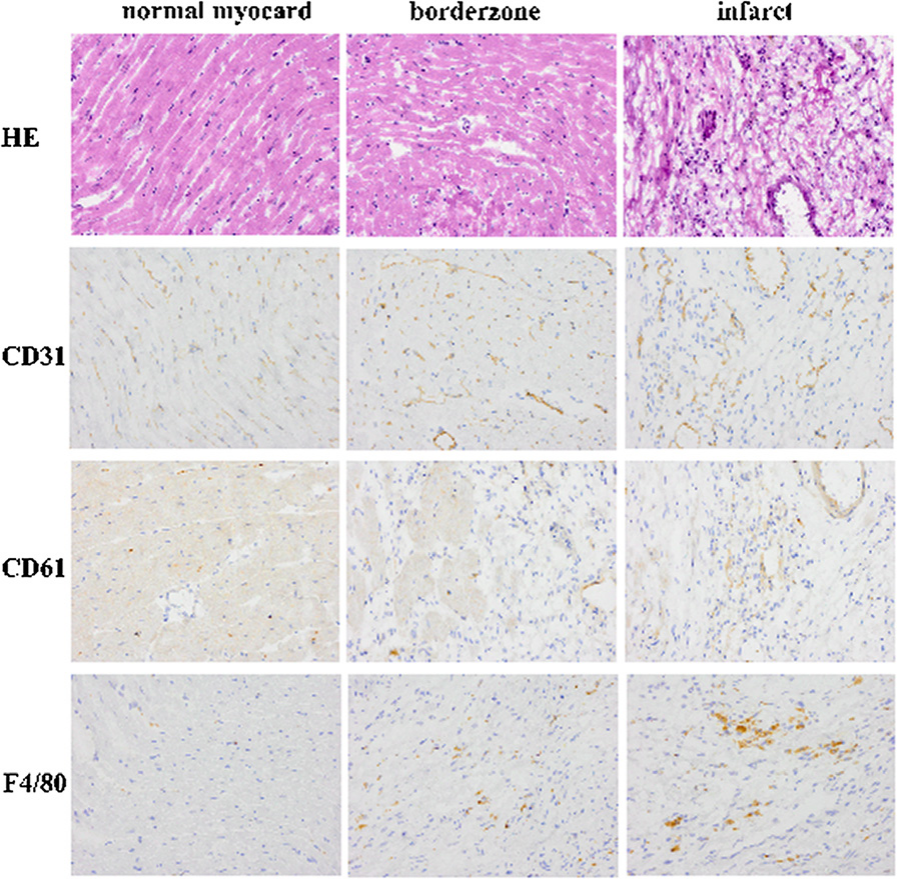
**Representative myocardial sections.** HE, CD31 (endothelial cells), CD61 (β_3_ integrin) and F4/80 (macrophages) staining in infarct, border zone and remote areas. Original image ×400 magnification.

Immunohistochemical staining showed increased density of vessels, macrophages and β_3_ integrin in the border zone (Figures 1 and 2). The sprouting vessels in the myocardial infarct showed upregulation and expression of β_3_ integrin, whereas only a faint expression in the muscle cells in the remote myocardium was observed (Figure 2). There were no differences between groups on the vascular density (72 ± 5 vs. 75 ± 5), macrophages (15 ± 6 vs. 17 ± 5) or CD61 positive cells (63 ± 4 vs. 64 ± 6, for the ^68^Ga-NODAGA-RGD and ^68^Ga-TRAP(RGD)_3_ groups, respectively). The size of the focal uptake area in the autoradiogram sections was comparable between the three groups (8.2 ± 5.1; 7.3 ± 3.2 and 7.4 ± 4.5 mm^2^ for ^18^F-galacto-RGD, ^68^Ga-NODAGA-RGD and ^68^Ga-TRAP(RGD)_3_, respectively).

### PET-CT *in vivo* imaging

**Figure 3:**
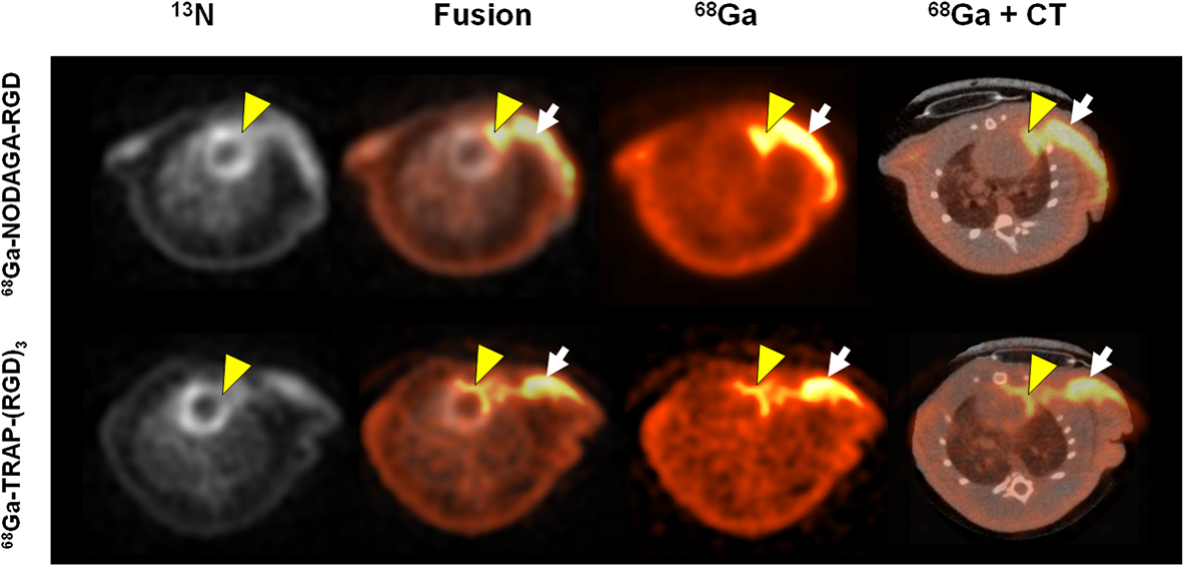
***In vivo *PET/CT images of rat MI.** Representative transaxial sections show hypoperfused myocardium area (^13^N) and corresponding RGD uptake (^68^Ga, % ID/g). The focal uptake is seen in infarct (yellow arrowheads) and the operation scar (white arrows), as verified by CT scan.

Augmented uptake of both ^68^Ga-RGD tracers co-localised to the [^13^N]NH_3_ area of hypoperfusion (low signal) in the myocardium as seen in the transversal axis images (Figure 3). A substantial uptake was seen also in the operation scar, which could be verified with co-registered PET and CT images. The image-derived average uptakes in the myocardium were 0.17 ± 0.03 and 0.25 ± 0.02% ID/cc for infarct areas at 82.5 min after injection for ^68^Ga-NODAGA-RGD (*n* = 4) and ^68^Ga-TRAP(RGD)_3_ (*n* = 3), respectively. Compared to the uptake in the remote myocardium, the infarct-to-remote ratios were 2.1 for ^68^Ga-NODAGA-RGD and 1.9 for ^68^Ga-TRAP(RGD)_3_.

### *Ex vivo* biodistribution

**Figure 4:**
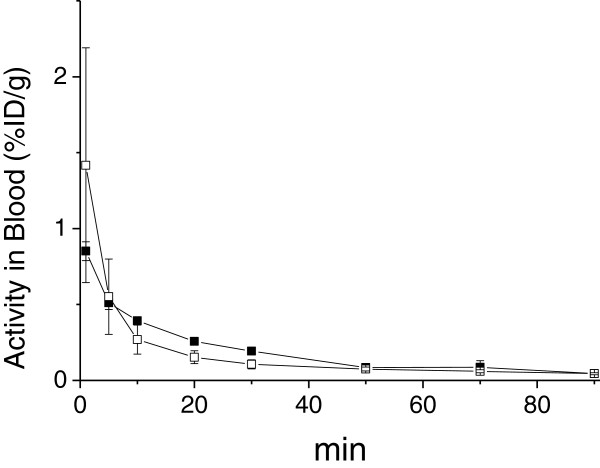
**Blood clearance as measured by gamma counting.** Closed symbols ^68^Ga-NODAGA-RGD, open symbols ^68^Ga-TRAP(RGD)_3_.

The accumulated activity of ^68^Ga in tissues as measured with gamma counting is presented in Table 1. The highest ^68^Ga activities were seen in the urine and kidney, indicating renal excretion as previously reported in mice [10-12]. The uptake values were comparable, independent of the weight of the animal or injected dose, with exception of the healthy connective tissue, muscle and remote myocardium where ^68^Ga-TRAP(RGD)_3_ (*n* = 10) uptake was found to be higher compared to ^68^Ga-NODAGA-RGD (*n* = 9). The blood time activity curves showed similar kinetics of all three tracers with rapid clearance from the circulation (Figure 4 and in [6]).

**Table 1:** **Biodistribution of **^**68**^**Ga-NODAGA-RGD and **^**68**^**Ga-TRAP(RGD)**_**3 **_**in tissues 90 min p.i**

	^**68**^**Ga-NODAGA-RGD**	^**68**^**Ga-TRAP(RGD)**_**3**_	
	**Mean ± SD**	**Mean ± SD**	
	***n *= 9**	***n *= 10**	***p *Value**
Blood	0.058 ± 0.02	0.101 ± 0.07	ns
Serum	0.094 ± 0.05	0.166 ± 0.12	ns
Remote myocardium	0.071 ± 0.03	0.154 ± 0.09	0.02
Apical myocardium	0.156 ± 0.06	0.353 ± 0.10	-
Muscle	0.035 ± 0.01	0.066 ± 0.03	0.028
Connective tissue	0.110 ± 0.05	0.190 ± 0.08	0.031
Wound	0.270 ± 0.24	0.256 ± 0.12	ns
Spleen	0.246 ± 0.16	0.415 ± 0.30	ns
Liver	0.230 ± 0.17	0.524 ± 0.61	ns
Kidney	1.102 ± 0.70	1.447 ± 0.51	ns
Urine	20.24 ± 6.40	35.10 ± 22.37	ns

Ns, not significant; p.i., post-injection. Results presented as percentage of injected dose per gram of tissue (% ID/g). The apical myocardium contains both infarct and remote areas, as can be seen in processed transaxial sections for autoradiography.

The apical part of the myocardium, containing both infarcted and remote myocardial tissue, was measured before processing into sections. The uptake was higher in the apical part as compared to the remote myocardium tissue sample (apical-to-remote ratio 2.4 (*p* > 0.01) for ^68^Ga-NODAGA-RGD and 2.1 (*p* > 0.01) for ^68^Ga-TRAP(RGD)_3_).

### *Ex vivo* autoradiography of RGD tracer uptake in the myocardium

**Figure 5:**
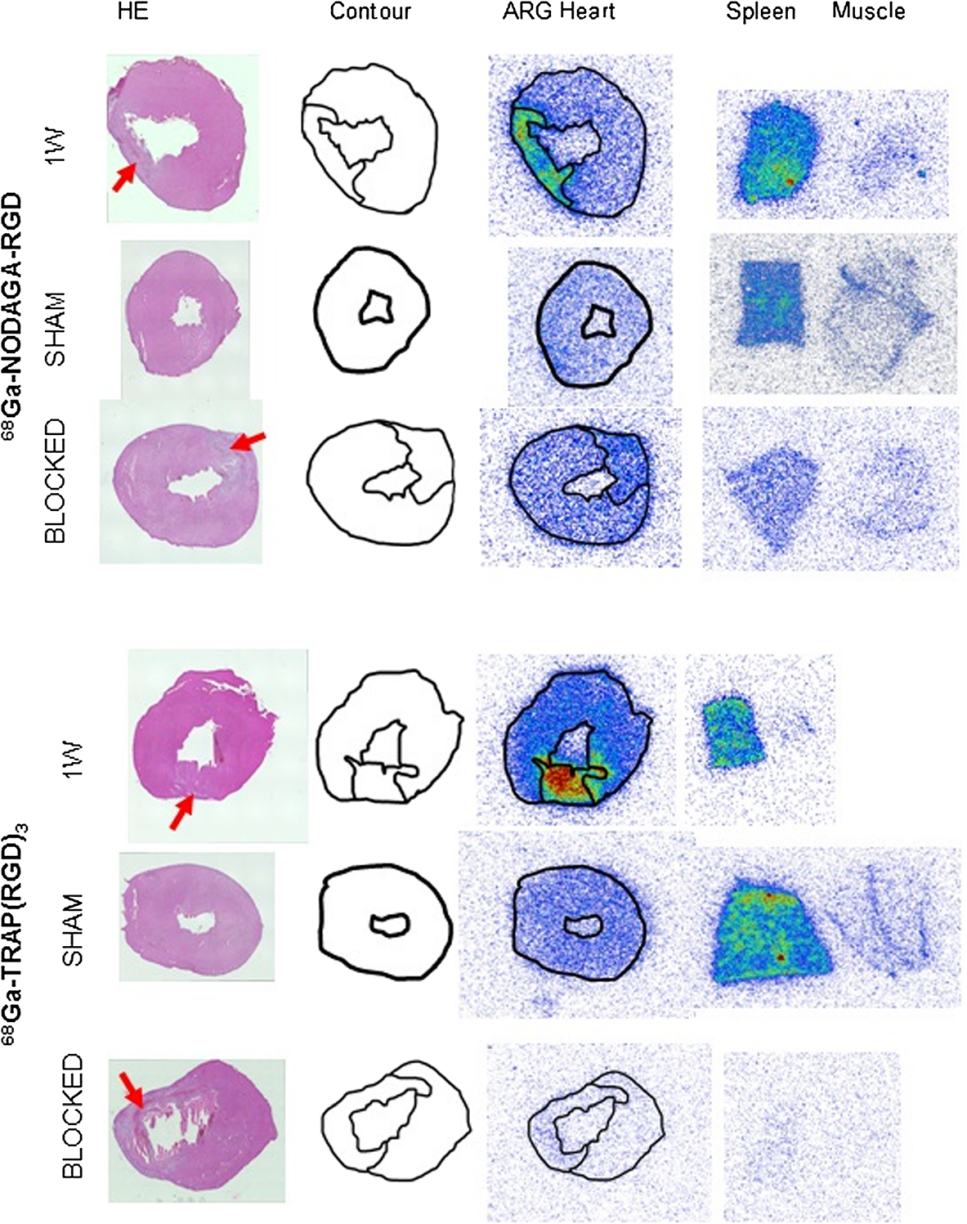
**Autoradiograms of myocardial cross sections. **^68^Ga-NODAGA-RGD and ^68^Ga-TRAP(RGD)_3_ uptake at 1 week after MI, in sham-operated controls and with a pre-injection of blocking dose of cilengitide. From left to right: HE-stained 20-μm section, delineated infarct area (arrow, contour), digitised autoradiogram of the same section where focal uptake matches infarct area. Digitised autoradiogram of 20-μm sections of the spleen (positive control) and muscle (negative control), with the same colour scale.

**Figure 6:**
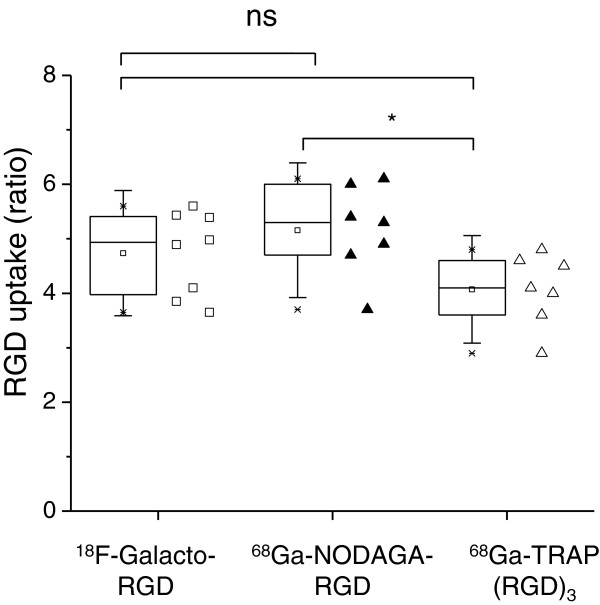
**RGD uptake ratio in MI autoradiograms.** Mean infarct to remote ratio of PSL/mm^2^ and SD of three tracers.

In autoradiography images, an augmented uptake of all three tracers was observed within the infarct area as verified by HE staining (Figure 5). The infarct vs. remote PSL/mm_2_ uptake ratios were 4.7 ± 0.8, 5.2 ± 0.8 and 4.1 ± 0.7 for ^18^F-galacto-RGD (*n* = 8), ^68^Ga-NODAGA-RGD (*n* = 7) and ^68^Ga-TRAP(RGD)_3_ (*n* = 7), respectively (Figure 6). The ^68^Ga-NODAGA-RGD infarct-to-remote ratio was higher as compared to ^68^Ga-TRAP(RGD)_3_ (*p* = 0.04), but neither of the ^68^Ga tracers differed from ^18^F-galacto-RGD (*p* > 0.05).

The tracer distribution in the myocardium of the sham-operated rats was uniform and comparable to the remote area of the operated rats (Figure 5). Pre-injection of high-dose cilengitide [12-15] reduced the uptake in the myocardium and also in the spleen, demonstrating specific binding (Figure 5). Infarct-to-remote ratio was 1.7 for both tracers when blocked with cilengitide (*n* = 3).

**Figure 7:**
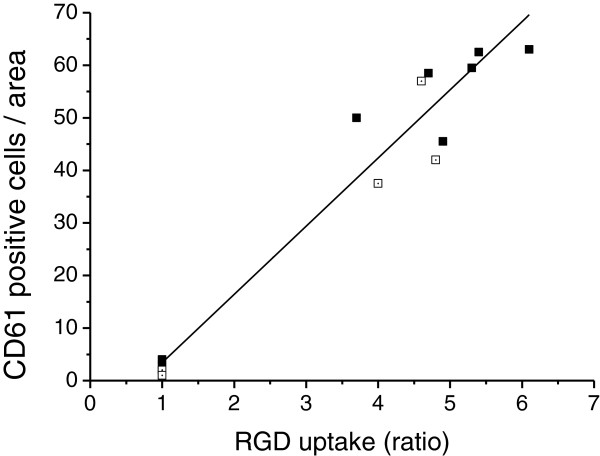
**Correlation of β**_**3 **_**integrin expression (CD61) and RGD uptake.** Pooled results from autoradiography ratios of ^68^Ga-NODAGA-RGD (closed symbols) and ^68^Ga-TRAP(RGD)_3_ (open symbols) uptake (*R* = 0.91).

The RGD uptake in the infarct and borderzone area of the myocardium, normalised to the remote area, correlated with β_3_ integrin expression (*R* = 0.91) (Figure 7).

## Discussion

Studies in the rat model of myocardial infarct have shown that monitoring the expression of α_v_β_3_ integrin by molecular imaging provides valuable prognostic information about myocardial repair after infarct [5-8]. First studies with animal models and humans using ^18^F-galacto-RGD have been promising, but further studies using this tracer are limited due to the difficulties of producing ^18^F-galacto-RGD in compliance with the GMP. Therefore, we sought to study new ^68^Ga-labelled RGD tracers for the use for MI imaging. Using *in vivo* imaging and *ex vivo* autoradiography analysis, we show that ^68^Ga-NODAGA-RGD and ^68^Ga-TRAP(RGD)_3_ accumulate in the myocardial infarct area in a similar manner as ^18^F-galacto-RGD. Autoradiography analysis revealed 4.1 to 5.2 times higher uptake in the infarct area as compared to the remote myocardium. Both *in vivo* and *ex vivo* gamma counting results supported this finding, although attenuation, partial volume and signal spillage diminished the acquired infarct-to-remote ratios obtained using these methods. In comparison to ^18^F-galacto-RGD, neither of the ^68^Ga-labelled tracers was superior, but when compared between each other, ^68^Ga-NODAGA-RGD had slightly higher infarct-to-remote myocardium ratio in the autoradiograms. This was not affected by the infarct size, vascular density or macrophage count. Moreover, we found that the RGD uptake correlated with β_3_ integrin expression in the myocardium.

Integrins have been shown to be essential in maintaining the homeostasis of the myocardium after pathologic stressors, such as myocardial infarct injury [16]. Adverse healing of infarct leads to maladaptive hypertrophic growth of the left ventricle and, therefore, biomarkers for early detection are sought after. In addition to the role in the formation of neovessels during the granulation phase of infarct healing, the α_v_β_3_ integrin participates in signalling events for further processes. β_3_ integrin together with β_1_ integrin play a role in hypertrophic growth *via* the activation of survival mechanisms by attenuating myocyte apoptosis [16-19]. Therefore, molecular imaging of integrin expression may offer an attractive way to improve risk assessment of post-MI patient and may serve as a biomarker for targeted therapy.

Cyclic RGDfK pentapeptides have been extensively studied for α_v_β_3_ integrin imaging [20,21]. Cyclic RGDfK and RGDyK compounds have a high affinity in the nanomolar range to α_v_β_3_ and α_v_β_5_ and have selectivity over other integrin subtypes [22,23]. Several RGD compounds for PET and SPECT have been introduced for integrin imaging in malignant tumours and angiogenesis [24]. The potential of α_v_β_3_ targeting RGD peptides for imaging of myocardial infarct healing has also been demonstrated recently using ^18^F-galacto-RGD [6,8], ^99m^Tc-RAFT-RGD [25] and ^18^F-AlF-NOTA-PRGD_2_[7].

^68^Ga-NODAGA-RGD is a cyclic RGD pentapeptide conjugated with a NODAGA chelator without affecting the binding affinity. ^68^Ga-TRAP(RGD)_3_[12] is a trimer based on a TRAP chelator for gallium(III) binding [11,26,27]. The specificity of ^68^Ga-NODAGA-RGD and ^68^Ga-TRAP(RGD)_3_ has been demonstrated in cell culture and mouse models [10-12], as well as for ^18^F-galacto-RGD in the same animal model as used in this study [6,8]. We also show that pre-injection of a blocking dose of cilengitide, a cyclic RGD peptide with high affinity for α_v_β_3_ and α_v_β_5_ integrins [14,15], diminished the ^68^Ga-NODAGA-RGD and ^68^Ga-TRAP(RGD)_3_ signal in the infarcted area of the myocardium and in the other measured tissues such as spleen and muscle, showing that the signal in these tissues is specific to integrin expression.

The affinity of trimeric ^68^Ga-TRAP(RGD)_3_ to α_v_β_3_ integrin is more than seven times higher than that of the monomers ^68^Ga-NODAGA-RGD and ^18^F-galacto-RGD (IC_50_ of 44 nM vs. 336 nM and 319 nM, respectively, determined in a competitive displacement assay on M21 human melanoma cells [9,10]). Multimerisation of the RGD molecule has been shown to increase affinity [20,28,29], but not necessarily the tumour-to-background ratio. This can be due to the specific (blockable) uptake of the ‘background’ tissue, which may express small amounts of the integrin. Another explanation can be the relatively low local concentration of the integrins in the target area and hence lack of bivalency binding of a multimeric molecule [10,30,31]. We also found a similar effect: the infarct-to-remote ratio in myocardium was not increased in the case of ^68^Ga-TRAP(RGD)_3_. In this case, the increased affinity of ^68^Ga-TRAP(RGD)_3_ to the small amount of integrin present in remote myocardium [4] results in a higher overall uptake, as was seen *in vivo* and *ex vivo* when compared to ^68^Ga-NODAGA-RGD (Table 1).

The selective and specific binding of cyclic RGD molecules to α_v_β_3_ and α_v_β_5_ integrins has been established with *in vitro* methods as well as *in vivo* experiments (reviewed in [20,32]). The α_v_β_3_ integrin expression is upregulated in several cancer cell lines [33] and in sprouting and activated vessels during wound healing of the skin, muscle and myocardium [4,34,35]. In supporting the earlier data of integrin location [4], the recent study by Gao and co-workers used dual immunofluorescence staining to demonstrate dominant co-localisation of CD61 (β_3_) signal with CD31-positive endothelial cells at 1 week after experimental ischaemia-reperfusion-induced infarct [7]. We also show that RGD signal correlates with the number of CD61-positive cells in permanent ligation-induced MI. Previously, we have shown that ^18^F-galacto-RGD uptake does correlate to the vascular density in the infarct area, but does not correlate to the amount of macrophages at 1 week after injury [8]. At 3 days after myocardial ischaemia reperfusion injury, Gao and colleagues did not find a strong co-localisation of CD61 and CD31 [7], which could indicate that the other cells might be responsible for RGD signal at this earlier time point. Myofibroblasts also express α_v_β_3_ integrins and can be targeted with a pro-collagen-targeting Cy5.5-RGD imaging peptide [36]. In addition to neovessels and myofibroblasts, macrophages have also been shown to express α_v_β_3_ integrin [37]. However, the relative amounts of integrin in these cell types have not been compared over time in MI. The temporal changes of the expression of α_v_β_3_ and other integrins and, therefore, the cellular localization of the signal of labelled cRGD compounds in the case of myocardial infarct still warrant further studies.

Taken together, we as well as several other groups have established the use of integrin imaging in monitoring of myocardial infarct healing and the feasibility of cyclic RGD compounds for that purpose. The current requirements for the production of radiopharmaceuticals in GMP compliance have made the clinical use of ^18^F-galacto-RGD difficult. There is an increasing number of different RGD imaging compounds available, but which candidate should be selected further for clinical use is an open question. Although high specificity and selectivity to integrins and high affinity are necessary qualities, the selection for a clinically usable tracer needs to take into account the practical issues such as the ease and safety of the production. ^68^Ga tracers appeal to sites with no cyclotron close by, but for instance, the FASTlab production cassette for ^18^F-fluciclatide is also already available [38], which will facilitate the clinical use of this RGD-containing tracer using F-18 and a distribution network. Ultimately, the resulting imaging quality is a factor that cannot be compromised.

## Conclusions

Prominent *in vivo* and *ex vivo* signal of ^68^Ga-NODAGA-RGD and ^68^Ga-TRAP(RGD)_3_ was found in the myocardium of rats at 1 week post infarct, localising to the area of hypoperfusion and β_3_ integrin expression. Both ^68^Ga-NODAGA-RGD and ^68^Ga-TRAP(RGD)_3_ showed similar binding behaviour in the myocardium after infarct as ^18^F-galacto-RGD. These results show the potential of ^68^Ga-labelled cyclic RGD peptides to monitor angiogenesis after myocardial injury *in vivo*.

## Competing interest

The authors declare that they have no conflict of interest.

## Authors’ contributions

IL took part in the conception and design, did the acquiring, analysing and interpreting of the data and wrote the manuscript. JN, KP, SN and GH synthesised the precursors and radiotracers. JN and KP acquired and interpreted the data and revised the manuscript critically. MR took part in the conception and design, acquired, analysed and interpreted data. EF carried out the animal models and revised of the manuscript critically. SGN is also involved in the interpretation of the *in vivo* data and revised the manuscript critically. HK, HJW and MS took part in the conception and design, interpreting of the data and revision the manuscript. All authors read and approved the final manuscript.
